# Supplementary material for: Mammalian orthoreovirus can exit cells in extracellular vesicles
Source: bioRxiv. 2023 Aug 29:2023.08.29.555250. Preprint. [Version 1] doi: 10.1101/2023.08.29.555250 (PMC10491149; doi:10.1101/2023.08.29.555250)
Supplement: Supplement 1 [file NIHPP2023.08.29.555250v1-supplement-1.pdf]

bioRxiv preprint doi: https://doi.org/10.1101/2023.08.29.555250; this version posted August 29, 2023. The copyright holder for this preprint (which was not certified by peer review) is the author/funder, who has granted bioRxiv a license to display the preprint in perpetuity. It is made available under aCC-BY 4.0 International license.

SUPPORTING INFORMATION

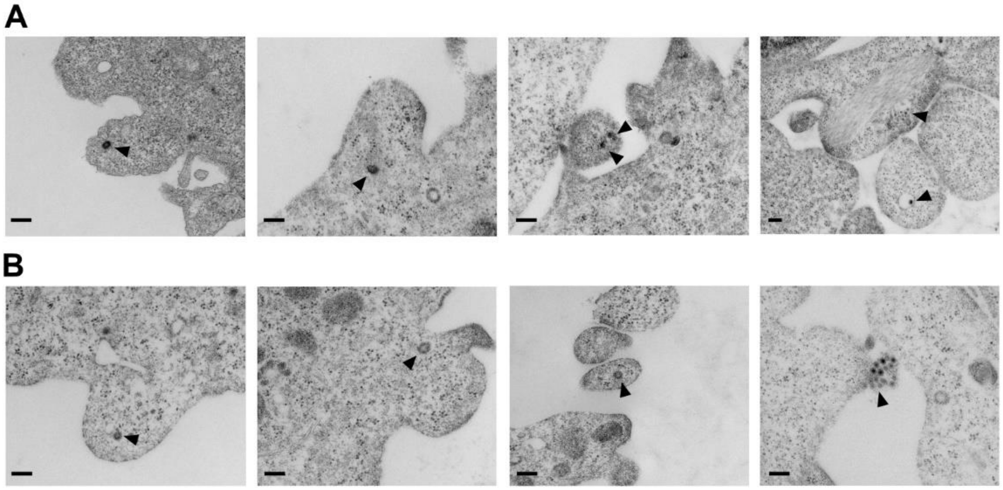

S1 Fig. EV-mediated reovirus egress is consistent with microvesicle biogenesis. Transmission electron microscopy of T1L-infected (A) or T3D-infected (B) L cells at 24 h p.i. Arrows point to viral particles observed near bleb-like structures budding from the plasma membrane in or around cells. Scale bar = 200 nm.

bioRxiv preprint doi: https://doi.org/10.1101/2023.08.29.555250; this version posted August 29, 2023. The copyright holder for this preprint (which was not certified by peer review) is the author/funder, who has granted bioRxiv a license to display the preprint in perpetuity. It is made available under aCC-BY 4.0 International license.

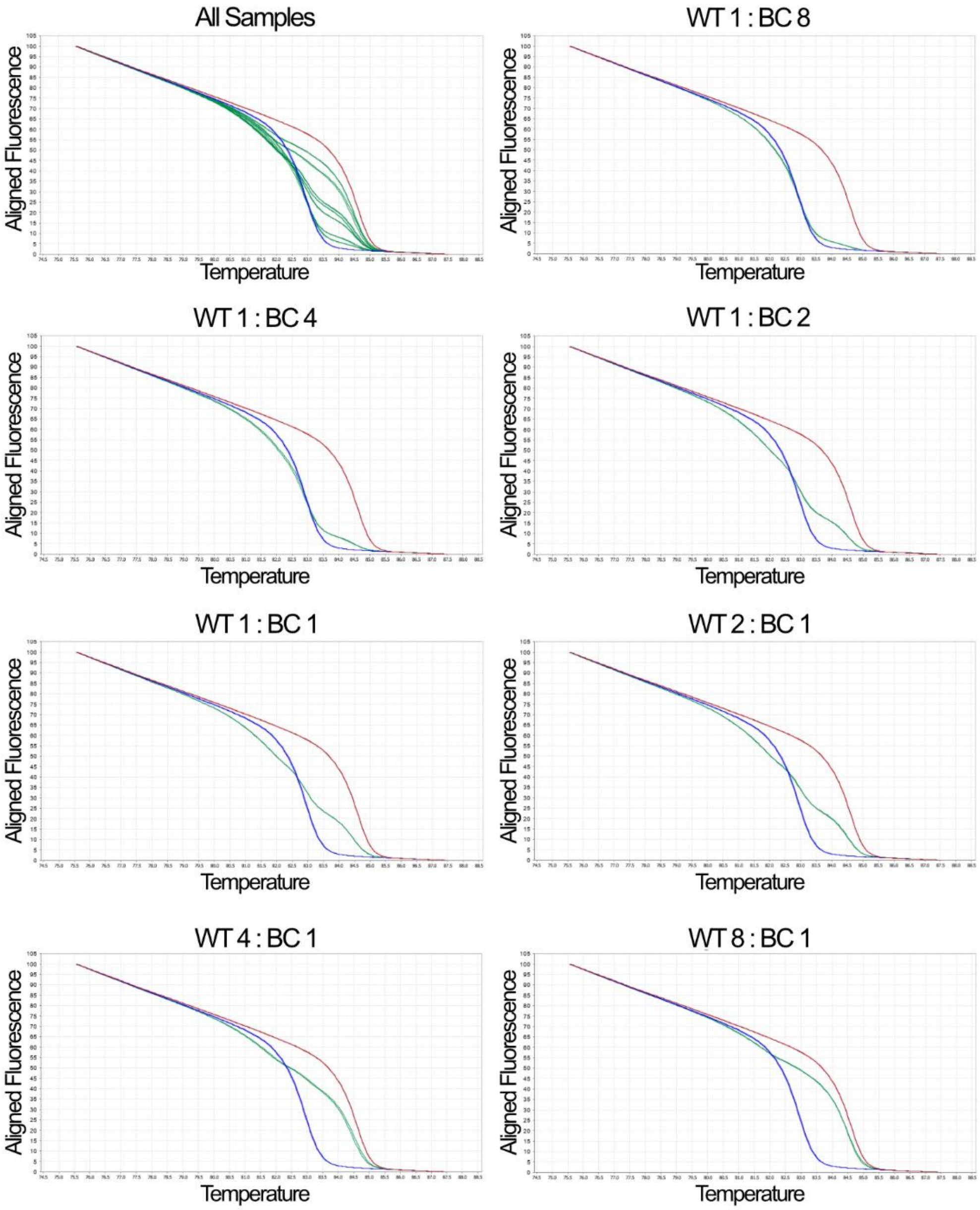

S2 Fig. Sensitivity of high-resolution melt analysis. Normalized melt curves for control RNA from WT (red), BC (blue), and mixtures of WT and BC (green) at the indicated ratios are shown.

bioRxiv preprint doi: https://doi.org/10.1101/2023.08.29.555250; this version posted August 29, 2023. The copyright holder for this preprint (which was not certified by peer review) is the author/funder, who has granted bioRxiv a license to display the preprint in perpetuity. It is made available under aCC-BY 4.0 International license.

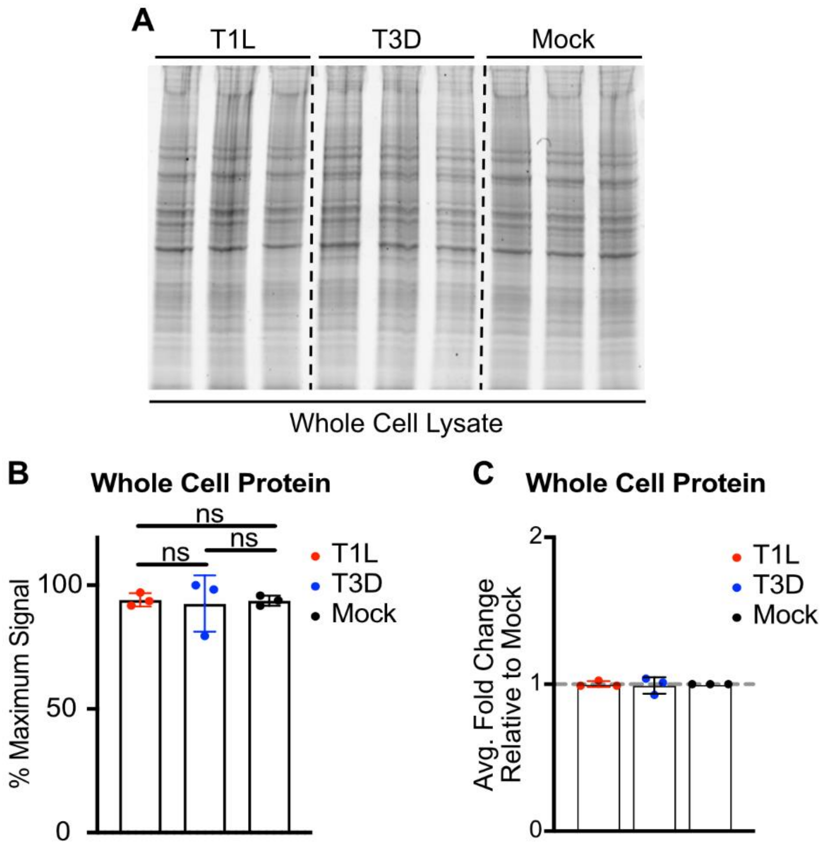

S3 Fig. Reovirus infection does not significantly alter whole cell protein expression. L cells were adsorbed with media (mock) or with three individual clones of T1L or T3D reovirus at an MOI of 1 PFU/cell for 72 h. (A-C) Cells were lysed in RIPA buffer, and lysates were resolved by SDS-PAGE and Coomassie staining (A), three independent experiments were quantified (B), and normalized by dividing the average virus-infected value by the average mock-infected value (C). Error bars indicate SD, n = 3. Comparisons by one-way ANOVA with Tukey's multiple comparisons.

bioRxiv preprint doi: https://doi.org/10.1101/2023.08.29.555250; this version posted August 29, 2023. The copyright holder for this preprint (which was not certified by peer review) is the author/funder, who has granted bioRxiv a license to display the preprint in perpetuity. It is made available under aCC-BY 4.0 International license.

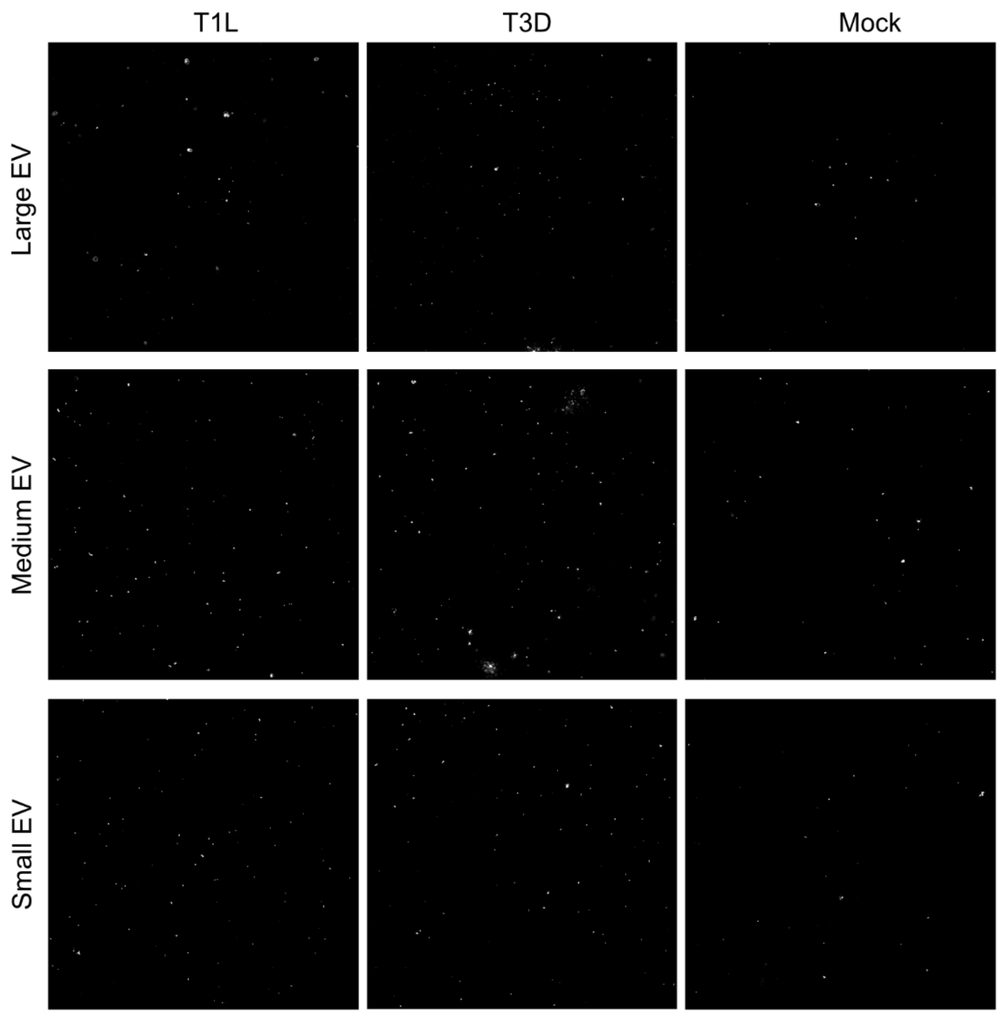

S4 Fig. Reovirus infection enhances EV release. Representative confocal images described in **Fig. 6G-H** are displayed for a single field of view, which is made up of an 8 x 8 tile imaging structure under 63X oil immersion.
